# Pro-Anorexia and Anti-Pro-Anorexia Videos on YouTube: Sentiment Analysis of User Responses

**DOI:** 10.2196/jmir.5007

**Published:** 2015-11-12

**Authors:** Atte Oksanen, David Garcia, Anu Sirola, Matti Näsi, Markus Kaakinen, Teo Keipi, Pekka Räsänen

**Affiliations:** ^1^ School of Social Sciences and Humanities University of Tampere Tampere Finland; ^2^ Chair of Systems Design ETH Zurich Zurich Switzerland; ^3^ Department of Social Research University of Turku Turku Finland

**Keywords:** eating disorders, anorexia, social media, emotions

## Abstract

**Background:**

Pro-anorexia communities exist online and encourage harmful weight loss and weight control practices, often through emotional content that enforces social ties within these communities. User-generated responses to videos that directly oppose pro-anorexia communities have not yet been researched in depth.

**Objective:**

The aim was to study emotional reactions to pro-anorexia and anti-pro-anorexia online content on YouTube using sentiment analysis.

**Methods:**

Using the 50 most popular YouTube pro-anorexia and anti-pro-anorexia user channels as a starting point, we gathered data on users, their videos, and their commentators. A total of 395 anorexia videos and 12,161 comments were analyzed using positive and negative sentiments and ratings submitted by the viewers of the videos. The emotional information was automatically extracted with an automatic sentiment detection tool whose reliability was tested with human coders. Ordinary least squares regression models were used to estimate the strength of sentiments. The models controlled for the number of video views and comments, number of months the video had been on YouTube, duration of the video, uploader’s activity as a video commentator, and uploader’s physical location by country.

**Results:**

The 395 videos had more than 6 million views and comments by almost 8000 users. Anti-pro-anorexia video comments expressed more positive sentiments on a scale of 1 to 5 (adjusted prediction [AP] 2.15, 95% CI 2.11-2.19) than did those of pro-anorexia videos (AP 2.02, 95% CI 1.98-2.06). Anti-pro-anorexia videos also received more likes (AP 181.02, 95% CI 155.19-206.85) than pro-anorexia videos (AP 31.22, 95% CI 31.22-37.81). Negative sentiments and video dislikes were equally distributed in responses to both pro-anorexia and anti-pro-anorexia videos.

**Conclusions:**

Despite pro-anorexia content being widespread on YouTube, videos promoting help for anorexia and opposing the pro-anorexia community were more popular, gaining more positive feedback and comments than pro-anorexia videos. Thus, the anti-pro-anorexia content provided a user-generated counterforce against pro-anorexia content on YouTube. Professionals working with young people should be aware of the social media dynamics and versatility of user-generated eating disorder content online.

## Introduction

Over the last decade, the emergence of pro-anorexia (ie, pro-ana) online communities has become a growing public health concern. With the help of information technologies, such communities are easily accessible and interactive, while encouraging harmful weight loss and weight control practices [1-6]. According to a 25-country EU Kids Online survey, 10% of children aged 9 to 16 years had seen eating disorder sites online, with girls being more commonly exposed to such material than boys [7]. Those exposed to such sites display a higher drive for thinness and perfectionism as well as a more negative perception of their appearance [8-10]. In addition, members of pro-anorexia communities report high levels of disordered eating [2,6,11]. Pro-anorexia communities have become a public concern and have aroused critical responses in social media, one example being so-called anti-pro-anorexia (ie, anti pro-ana) communities which oppose pro-anorexia content and provide support for those who are recovering from anorexia [3,12]. This is the first study of pro-anorexia communities that analyzes a large sample of data with sentiment analysis software. It provides a new perspective on the pro-anorexia phenomenon by analyzing emotional reactions to pro-anorexia and anti-pro-anorexia content on YouTube.

The rationale for the use of sentiment analysis here lies in the rapid expansion of social media content. YouTube has more than 1 billion users with 300 hours of video being uploaded every minute [13]. One of the central social media features of YouTube is the opportunity for those watching the videos to either like or dislike the video or to post a textual comment in the comment section below the video. These options are available for all registered users. This interactive feature of YouTube provides insight into audience reactions that can help us to understand how, for example, pro-anorexia videos are reacted to collectively. The social arena provided by YouTube is content driven and, as such, interaction is based on audience participation (eg, through commenting). The bonds formed over shared interests relayed by the videos in question vary in strength, some representing casual interest-based participation and others founded on more fundamental shared interests [14].

Those users commenting on videos concerning a shared interest can be considered part of a self-selected online community whose starting point is the online content itself. Online communities are defined as groups of individuals that interact through an online medium, regardless of the existence of explicit friendship links [15,16]. This concept is often applied to YouTube [17], where the most salient communication mechanism is through video comments. On YouTube, users do not necessarily establish “friendship” links and then subscribe to channels and comment on videos. In this setting, users interact via spontaneous discussion and reactionary expression through video comments rather than with established social peers as is more common on platforms such as Facebook or Twitter. Interaction between users on YouTube is indirect and delayed through textual comments or videos, yet this kind of delayed interaction is able to arouse collective emotions [15]. Furthermore, emotional expressiveness has been found to both motivate user participation and sustain online communities in the long term [16]. In the context of eating disorders, the audience response becomes important given that peer groups have an influence on disordered eating among adolescents [18,19]. Positive comments to pro-anorexia groups might increase their attractiveness and make anorexia more normalized.

Pro-anorexia communities are found on various social media sites, including Facebook [12], YouTube [20], Twitter, Instagram, Pinterest [21], and Flickr [22]. A new understanding of the scope of their activity requires comparing them to contrasting groups, such as anti-pro-anorexia communities. YouTube users commonly engage in active discussion by expressing either positive or negative sentiments in their messages [23]. User-generated anti-pro-anorexia content may, therefore, contest pro-anorexia content. Notably, responses within the YouTube community might be important for adolescents who tend to seek justification for their actions from peers rather than adults. Simultaneously, young people may also internalize certain values and behavioral norms more effectively from online communities than from various offline sources. These issues suggest that user-generated online content can be a significant source of influence. For example, a recent study found that pro-anorexia YouTube videos were more favored by viewers compared to informative videos describing the health consequences of anorexia [20].

In this study, we examined emotional reactions that pro-anorexia and anti-pro-anorexia videos received on YouTube among registered users. YouTube was selected for this study because it is the most popular social media site characterized by publicly available videos and comments. Both easy access and popularity make YouTube a significant source for information concerning anorexia. The study is built around 3 research questions: (1) what are the general characteristics of the pro-anorexia and anti-pro-anorexia videos and video uploaders, (2) what is the strength of both positive and negative emotional feedback received by these videos, and (3) how do the videos’ background information (eg, upload time, video length) associate with the comments posted?

## Methods

### User Profile Selection and Data Collection

The user profile selection was conducted October 15-29, 2014. During that time period, we retrieved YouTube videos using 2 search terms, namely “pro-ana” and “anti pro-ana.” Based on Google Trends queries, these were the most popular words used to describe the positive and negative stance toward anorexia. Using additional synonyms would not have changed the search results because of the YouTube search engine’s use of query expansion, which involves applying synonyms of search terms to increase recall. We received similar results using “pro-ana,” “proana,” and “pro-anorexia” and their “anti” counterparts with more than 90% coverage. These search terms were able to cover part of “thinspiration,” “thinspo,” “anti-thinspiration,” and “anti-thinspo” hits as well.

We selected the most popular profiles that included uploaded video material from the past 24 months concerning either the pro-anorexia or anti-pro-anorexia stance on anorexia, limiting both profile lists to 25 on the basis of their popularity. The popularity of profiles selected was determined by both video views and channel subscriptions. Notably, the YouTube search engine is also likely to list these channels first due to these criteria. All of the selected profiles were in English as were the included video comments posted during the previous 24 months. In addition, we checked that the profiles were either pro-anorexia or anti-pro-anorexia. The most popular material was created by individuals who often included video blogs concerning their opinion on anorexia. Our data did not include profiles that might be considered official profiles of governmental or nongovernmental organizations.

The second phase of data collection involved the retrieval of information about videos and comments through an automatic crawler using the YouTube Data Application Programming Interface [24]. On November 3-14, 2014, we extracted all of the video comments provided by YouTube as well as any available information of the videos, including title, date, description, and number of views, likes, and dislikes. This approach was similar to that of previous research on YouTube analyses of political campaigns [17] and video popularity [25]. The total video comment material included 1163 videos uploaded by 50 YouTube users. Although all of the users hosted video content concerning anorexia, some included a variety of other types of content. We first excluded 671 videos that did not include anorexia content. In addition, 97 videos were omitted because they did not have any comments. Hence, 395 videos with a total of 12,161 comments were included in the final sample.

User information included the date when the user joined YouTube, their physical location (country), and the number of subscribers to their channel. The gender of profile users was checked separately by viewing profile pages and videos (1=female; 2=male; 3=other). Video information included upload time, duration, number of video views, numbers of video likes and dislikes, and the total number of video comments and commenter usernames.

Video comments were assessed with the SentiStrength automatic sentiment analysis tool, which uses an algorithm to estimate the sentiment content of texts based on lexical information consisting of a list of sentiment words (approximately 3000) and grammatical categories (eg, negation) [26-28]. SentiStrength is particularly useful when working with big data and short comments, such as those of Twitter or YouTube, because it extracts both negative and positive sentiment strength simultaneously from a given short text, enabling the analysis of texts expressing both positivity and negativity at the same time. Notably, research on linguistics and psychology both show that textual statements can have loadings in both negative and positive scales simultaneously [28,29]. Positive ratings vary from 1 (not positive) to 5 (extremely positive) and negative ratings vary from -1 (not negative) to -5 (extremely negative).

### Reliability Tests

We ran interrater reliability checks throughout the data collection period. Interrater reliability of the YouTube profile users and video contents were assessed by 2 independent raters. Average interrater agreement was 90.68% (Cohen’s κ=.80).

The reliability of the SentiStrength tool was tested in the study. We ran the test with a random sample of 1000 comments with 3 blind reviewers who were given instruction to code the comments on both the positive and negative axis. The raters had, on average, 47.03% (470/1000) full agreement with SentiStrength on the positive scale and 63.70% (637/1000) on the negative scale. In all, 87.20% (872/1000) of their answers showed high similarity (ie, within 1 point of SentiStrength rating) on the positive scale and 88.30% (883/1000) on the negative scale. Mean correlation between raters and SentiStrength was ρ=.63 on the positive scale and ρ=.69 on the negative scale.

**Table 1:** Interrater reliability figures between SentiStrength and human raters.

Reliability figures^a^		SentiStrength	Rater 1	Rater 2	Rater 3
**Positive scale**					
	Mean (95% CI)	2.16 (2.10, 2.22)	2.11 (2.05, 2.18)	2.09 (2.03, 2.15)	2.74 (2.65, 2.82)
	Full agreement, %		53.60	55.60	31.90
	Close agreement, %		92.50	93.20	75.90
	Cohen’s κ		.349	.375	.139
	Spearman ρ		.635	.647	.596
**Negative scale**					
	Mean (95% CI)	–1.93 (–2.00, –1.85)	–1.75 (–1.82, –1.69)	–1.72 (–1.79, –1.66)	–1.94 (–2.01, –1.86)
	Full agreement, %		64.70	67.60	58.80
	Close agreement, %		89.70	91.00	84.10
	Cohen’s κ		.430	.470	.342
	Spearman ρ		.719	.728	.630

^a^ A random sample of 1000 comments were reviewed. Full agreement means that the human rater and SentiStrength had exactly the same rating. Close agreement means that the difference was maximum 1 point on the 5-point scale.

Table 1 shows means and 95% confidence intervals for SentiStrength and raters. It also shows the full agreement, close agreement, Cohen’s kappa, and Spearman rho figures for each rater. Based on a 2-sample Mann-Whitney *U* test, the difference between SentiStrength and raters 1 and 2 was not statistically significant on the positive scale (rater 1: *Z*=1.43, *P*=.15; rater 2: *Z*=1.90, *P*=.06). However, this difference was significant between SentiStrength and rater 3 (*Z*=–10.27, *P*<.001). On the negative scale, there was no statistically significant difference between SentiStrength and rater 3 (*Z*=–0.63, *P*=.53). However, raters 1 and 2 were statistically different on the negative scale (rater 1: *Z*=–2.80, *P*=.005; rater 2: *Z*=–3.63, *P*<.001). Overall, our results indicate that SentiStrength’s precision is within human-level accuracy in estimating sentiments from text.

### Measures and Statistical Methods

Our key interest was to analyze responses to both pro-anorexia videos and anti-pro-anorexia videos. First, we provided descriptive statistics on both video types. These included a Mann-Whitney 2-sample *U* test for the comparison of pro-anorexia and anti-pro-anorexia communities. Our main analyses were based on ordinary least squares (OLS) regression models. We analyzed both positive and negative feedback to the videos by investigating the content of comments and video likes and dislikes. Both negative and positive SentiStrength scales as well as video likes and dislikes were used as dependent variables.

Our regression models controlled for the number of video views, number of comments, number of months the video had been available on YouTube, and video duration. In light of the skewed distribution of these variables, we used logarithmic transformation (natural logarithm) in the analysis. We also controlled for the country of the video uploader (dummy coded, 0=non-English speaking, 1=English speaking) and activity of the uploader as a commentator (0=no; 1=yes). The models predicting positive and negative sentiments were adjusted to account for the clustering of observations on the level of videos. This procedure had an impact on standard errors. Therefore, we report the adjusted predictions on the regression models (with the other variables in the model set at their means) for both positive and negative sentiments as well as for video likes and dislikes.

## Results

### Video Uploader Statistics

Of 50 uploaders, 46 were female (92%) and the rest represented the category of unknown or “other” gender (eg, transgender). The uploaders came from 13 different countries, almost half from the United States (44%, 22/50); 74% (37/50) were from English-speaking countries. Their user profiles were, on average, 4 years old (mean 52.87 months, SD 28.23, range 9-102) and they had a mean of 2208 subscribers (SD 10,298.54, range 0-70,285). Uploaders were not active in writing comments to other videos. In addition, pro-anorexia profile users and anti-pro-anorexia profile users did not interact in our dataset. There were only 2 comments written by a pro-anorexia uploader to anti-pro-anorexia videos, whereas there were only 4 comments vice versa.

### Video Comment Statistics

Our data included 133 pro-anorexia videos and 262 anti-pro-anorexia videos. As Table 2 shows, these 395 videos had a total of 12,161 comments from 7903 commenters. Only 1.00% (79/7903) of the commenters commented on both pro-anorexia and anti-pro-anorexia videos. Uploaders wrote only 5.80% (705/12,161) of the comments. The videos had more than 6 million total views indicating that this kind of material is widely accessed by YouTube users. Videos had a mean of 15,496 views (SD 37,865.37, range 57-280,253). Table 2 also shows that anti-pro-anorexia videos were more popular based on the number of video views, comments, and commenters. Anti-pro-anorexia videos received more likes than pro-anorexia videos (*Z*=–3.21, *P*=.001) and they also received more positive comments based on SentiStrength analysis (*Z*=–3.78, *P*<.001).

**Table 2:** Descriptive statistics on pro-anorexia and anti-pro-anorexia videos on YouTube.

Video characteristics		Pro-anorexia	Anti-pro-anorexia
Videos, n		133	262
Comments, n		2114	10 047
Commenters, n		1594	6309
Comments/video		15.89	38.35
Comment uploaders, n		115	590
Video views (total), n		1.4 million	4.8 million
Videos views, mean		10,189	18,399
Video active (months), mean (SD)		34.16 (28.84)	18.63 (19.90)
Video duration (mins), mean (SD)		251.57 (178.71)	526.83 (404.09)
**Video likes/dislikes**			
	Likes, mean (SD)	33.56 (61.05)	179.03 (325.58)
	Dislikes, mean (SD)	7.35 (18.14)	7.20 (25.38)
**SentiStrength**			
	Positive	2.02	2.16
	Negative	–1.89	–1.89
	Positive (uploader)	1.75	1.92
	Negative (uploader)	–1.61	–1.75

#### Differences Between Responses to Pro-Anorexia and Anti-Pro-Anorexia Videos

Our OLS regression models were used to estimate the difference between responses to pro-anorexia and anti-pro-anorexia content after controlling for selected independent variables. The adjusted predictions are presented in Table 3. They show that there was a statistically significant difference between communities on both positive comments and video likes. Anti-pro-anorexia videos were commented on more positively and they also received significantly more video likes. For example, anti-pro-anorexia videos received an adjusted prediction (AP) of 181 (SE 13.81) likes compared to 31 (SE 3.36) likes for pro-anorexia videos. The difference between positive sentiments expressed in the video comments was also statistically significant. The analysis of negative sentiments and video dislikes showed that there were no statistically significant differences between pro-anorexia and anti-pro-anorexia videos.

**Table 3:** Adjusted predictions (APs)^a^ of positive and negative sentiments and video likes and dislikes for pro-anorexia and anti-pro-anorexia videos.

Sentiments and likes	Pro-anorexia, AP (95% CI)	Anti-pro-anorexia, AP (95% CI)
Positive sentiment (1 to 5)	2.02 (1.98, 2.06)	2.15 (2.11, 2.19)
Negative sentiment (-1 to -5)	–1.89 (–2.00, –1.77)	–1.89 (–1.94, –1.84)
Video likes	31.22 (24.62, 37.81)	181.02 (155.19, 206.85)
Videos dislikes	7.30 (5.05, 9.55)	7.31 (4.44, 10.18)

^a^ APs are based on OLS regression models that controlled for the number of video views and comments, number of months the video had been on YouTube, the duration of the video, uploader’s activity as a video commentator and uploader’s country information.

**Table 4:** Ordinary least squares (OLS) regression models^a^ on positive and negative sentiments and video likes and dislikes for pro-anorexia and anti-pro-anorexia videos.

Video characteristics		Positive		Negative		Likes		Dislikes	
		*B*	*P*	*B*	*P*	*B*	*P*	*B*	*P*
**Pro-anorexia** ^b^									
	Video views	–0.12	<.001	0.05	.45	17.85	<.001	6.98	<.001
	Number of comments	0.06	.087	–0.20	<.001	11.48	.005	0.56	.68
	Time available online	0.18	<.001	0.01	.80	–14.58	.002	–5.91	<.001
	Video duration	0.07	.04	0.10	.15	5.30	.26	–1.33	.40
	Uploader commented video	–0.34	<.001	0.16	.17	2.64	.85	11.68	.01
	Uploader (English-speaking country)	–0.04	.47	–0.18	.15	9.33	.38	10.74	.004
	Constant	1.93	<.001	–2.09	.001	–109.14	.002	–23.69	.045
**Anti-pro-anorexia** ^c^									
	Video views	–0.13	<.001	0.05	.06	89.31	<.001	3.42	.02
	Number of comments	0.08	.006	–0.08	.02	66.34	<.001	2.43	.18
	Time available online	–0.07	.049	0.03	.36	–55.61	.003	–1.35	.52
	Video duration	0.10	.005	0.12	.01	–17.76	.45	–3.32	.20
	Uploader commented video	–0.40	<.001	0.11	.06	–15.04	.71	1.24	.78
	Uploader (English-speaking country)	0.00	.98	0.14	.03	64.47	.08	3.21	.42
	Constant	2.77	<.001	–2.89	<.001	–475.99	.01	–4.17	.84

^a^ *B* refers to the unstandardized regression coefficients. Uploader as commenter and country are included in the model as dummy variables. Logarithmic transformation was used for other independent variables. Range of positive from 1 (not positive) to 5 (extremely positive). Range of negative from –1 (not negative) to –5 (extremely negative).

^b^ For pro-anorexia, positive model n=2114, negative model n=2114, likes model n=133, and dislikes model n=133.

^c^ For anti-pro-anorexia, positive model n=10,000, negative model n=10,000, likes model n=259, and dislikes model n=259.

Our OLS regression models (Table 4) showed that video background information was significantly associated with the positive axis (positive sentiments and video likes), but less with the negative axis (negative sentiments and video dislikes). These findings are important in understanding the expressed sentiments. Highly accessed pro-anorexia and anti-pro-anorexia videos were more likely to receive less favorable comments (*B*=–0.12, *P*<.001 and *B*=–0.13, *P*<.001, respectively). Longer videos gained more positive comments. Furthermore, the videos were less likely to receive positive sentiments if their uploader had commented on them. Country did not have any impact on comments. The number of anti-pro-anorexia video comments was associated with positive sentiments. On the negative axis, the number of comments was likely to increase the negativity of the comments for both pro-anorexia and anti-pro-anorexia videos (*B*=–0.20, *P*<.001 and *B*=–0.08, *P*=.02, respectively).

## Discussion

Our aim was to provide new insights into user reactions regarding pro-anorexia and anti-pro-anorexia content on YouTube using a sentiment analysis approach. We found that anti-pro-anorexia video comments expressed more positive sentiments than those of pro-anorexia videos. They also had more views and a higher number of video likes than pro-anorexia videos. Our analysis based on negative sentiments and video dislikes showed that there were no statistically significant differences between pro-anorexia and anti-pro-anorexia videos. Consistent with earlier findings showing that negativity is the fuel of online conversation [16,30], we also found that the number of video comments was associated with higher comment negativity. However, our analysis showed that major differences exist between responses to pro-anorexia and anti-pro-anorexia videos on the positive axis. Notably, in online commentaries, positive comments may facilitate affiliations between users, whereas negative comments are likely to be more complicated because emotional responses involving sadness and anger might provoke very different responses [16]. This may explain why we found differences on the positive axis, but not on the negative one.

Our results showed that there was little interaction between pro-anorexia and anti-pro-anorexia video commenters, with only 1% of users having commented on both pro-anorexia and anti-pro-anorexia videos. Similarly, a comparison of pro-anorexia and pro-recovery groups on Flickr showed that most of the interaction took place within each group [22]. However, in our study the videos were commented on by almost 8000 people, a number which reflects the popularity of YouTube. Within this context, anti-pro-anorexia uploaders gained higher visibility for their videos and, based on the positive sentiments and video likes, they were more effective in communicating their message. Earlier studies have shown that pro-anorexia communities are easily accessible [1,4] and well organized [12], and that their videos receive more likes than purely informative health videos [20]. In our case, anti-pro-anorexia videos were more popular, received more video likes, and, most importantly, they were commented on more positively. We believe that this difference is explained by the fact that in our study, anti-pro-anorexia videos were user generated and also represented recovering anorexics. As such, they provided an effective strategy to contribute to eating disorder recovery online.

Our results also deviate from a recent Facebook study in which a pro-anorexia group was found to be more active and better organized than an anti-pro-anorexia group [12]. The difference between these findings can be potentially explained by the differences between the types of social media examined, namely YouTube and Facebook. YouTube is a publicly available global platform for distributing content. Facebook, on the other hand, tends to provide a more personalized and structured platform that can be managed with greater detail. On Facebook, it is often an individual user that sets up the group and, thus, has more power and means to manage what is said and distributed within the group and how it is structured. This content is also commonly available only to those users who join the group. These are characteristics that can affect both the level of activity and top-down management of the group. On the other hand, YouTube material is available for all the users and it may spread virally to other YouTube users and other social media [31]. For example, the antivaccination movement was able spread information via YouTube and it publicly challenged the mainstream medical point of view [32]. Hence, we have to acknowledge the role and manipulative power associated with social media because it has been found to influence the type of news and information users consume [33].

### Limitations

Our study has its limitations. First, we examined only communities and videos within YouTube; therefore, the results cannot be generalized to other online communities that may function differently depending on varying social media user interfaces. Second, our work is limited to English-language content because it was used as a selection criterion. Thus, results might not apply to videos and comments written in other languages. Third, we limited our analysis to 50 uploaders who were selected based on their popularity. However, despite limiting ourselves to 50 users, the analysis involved a total of 1163 videos, representing a large dataset through limited sources. Fourth, our study focused on 2 contrasting search words that were most commonly used to express either a “pro” or “anti” statement toward anorexia. Despite this limited search criteria, the YouTube search engine optimization guaranteed coverage of synonyms. Thus, our search results are not compromised by using only 2 search words.

### Implications

Social media has been recognized as a valuable medium for health behavior identification and communication with adolescents and young adults [34]. One specific upside of this is its allowance for engaging so-called hard-to-reach populations [35]. Health and mental professionals working with young people would benefit from understanding social media communities as a whole. Although pro-anorexia content is now available in multiple social media sites [21], some are massively popular, such as YouTube. It is important to increase awareness of social media’s relevance among clinicians and educators [21,36]. Because YouTube does not restrict the material shown unless copyrights are broken, different censorship measures cannot be taken. Official routes may not be the most efficient in social media. Our results point out that YouTube users were able to respond to communities such as pro-anorexia and, in fact, those videos opposing pro-anorexia communities were more positively commented on and rated. A recognition of the dynamics of social media is important in understanding what groups or communities at-risk users associate with. This combination of dynamics and group identification would be important knowledge for any clinician treating eating disorders. Furthermore, the existing online material can be used for educational purposes. In addition, it would be possible to develop automatic tools based on thematic search and sentiment analysis to detect the most relevant online discussions on eating disorders in real time. Future studies should continue working with sentiments expressed on online social media platforms preferred by children and adolescents toward improved assessment of the effects of opposing points of view regarding health issues.

### Conclusion

Earlier studies have shown that pro-anorexia communities are active online and encourage unhealthy behavior [1-6]. Our study provides a more dynamic view of social media as we compared the YouTube user responses to both pro-anorexia and anti-pro-anorexia videos. The study showed that the pro-anorexia community has online opponents within the YouTube user community. Many anti-pro-anorexia videos were in fact pro-recovery videos. These videos promote help for anorexics and oppose the pro-anorexia community. These videos were also more popular, gaining more positive feedback and comments than pro-anorexia videos. Therefore, anti-pro-anorexia content appears to be a counterforce on YouTube. This study serves to benefit professionals working with young people by providing them with a deeper understanding of social media activities and the sentiments expressed therein.

## Abbreviations

AP: adjusted prediction
OLS: ordinary least squares
